# The Department of Veterans Affairs Gulf War Veterans’ Illnesses Biorepository: Supporting Research on Gulf War Veterans’ Illnesses

**DOI:** 10.3390/brainsci11101349

**Published:** 2021-10-14

**Authors:** Christopher B. Brady, Ian Robey, Thor D. Stein, Bertrand R. Huber, Jessica Riley, Nazifa Abdul Rauf, Keith R. Spencer, Gabriel Walt, Latease Adams, James G. Averill, Sean Walker, Ann C. McKee, Stephen P. Thomson, Neil W. Kowall

**Affiliations:** 1 Research and Development Service, VA Boston Healthcare System, Boston, MA 02130, USA; Jessica.Riley@va.gov (J.R.); Nazifa.AbdulRauf@va.gov (N.A.R.); krspencer1110@gmail.com (K.R.S.); gwalt@bu.edu (G.W.); Latease.Adams@va.gov (L.A.); 2 Department of Neurology, Boston University School of Medicine, Boston, MA 02118, USA; huberb@bu.edu (B.R.H.); nkowall@bu.edu (N.W.K.); 3 Harvard Medical School, Boston, MA 02115, USA; 4 Southern Arizona VA Healthcare System, Tucson, AZ 85723, USA; Ian.Robey@va.gov (I.R.); James.Averill@va.gov (J.G.A.); Sean.Walker@va.gov (S.W.); Stephen.Thomson@va.gov (S.P.T.); 5 Department of Endocrinology, University of Arizona, Tucson, AZ 85724, USA; 6 Pathology Service, VA Boston Healthcare System, Boston, MA 02130, USA; tdstein@bu.edu (T.D.S.); amckee@bu.edu (A.C.M.); 7 Department of Pathology and Laboratory Medicine, Boston University School of Medicine, Boston, MA 02118, USA; 8 Department of Veterans Affairs Medical Center, Bedford, MA 01730, USA; 9 National Center for Posttraumatic Stress Disorder, VA Boston Healthcare System, Boston, MA 02130, USA; 10 Neurology Service, VA Boston Healthcare System, Boston, MA 02130, USA

**Keywords:** gulf war, gulf war illness, chronic multisymptom illness, veterans, neuropathology, brain banking, tissue banking

## Abstract

Aims: To introduce a resource supporting research on Gulf War illness (GWI) and related disorders, the Gulf War Veterans’ Illnesses Biorepository (GWVIB). Methods: Gulf War era veterans (GWVs) are recruited nationally and enrolled via telephone and email/postal mail. Enrolled veterans receive annual telephone and mail follow-up to collect health data until their passing. A postmortem neuropathological examination is performed, and fixed and frozen brain and spinal cord samples are banked to support research. Investigators studying GWI and related disorders may request tissue and data from the GWVIB. Results: As of September 2021, 127 GWVs from 39 states were enrolled; 60 met the criteria for GWI, and 14 met the criteria for chronic multisymptom illness (CMI). Enrollees have been followed up to six years. Postmortem tissue recoveries were performed on 14 GWVs. The most commonly found neuropathologies included amyotrophic lateral sclerosis, chronic traumatic encephalopathy, and Lewy body disease. Tissue was of good quality with an average RNA integrity number of 5.8 (SD = 1.0) and ≥4.8 in all of the cases. Discussion: The availability of health data and high-quality CNS tissue from this well-characterized GWV cohort will support research on GWI and related disorders affecting GWVs. Enrollment is ongoing.

## 1. Introduction

About one-third of the nearly 700,000 men and women who served in Operations Desert Shield and Desert Storm during the 1990–1991 Gulf War (GW) have experienced chronic multisystem illnesses (CMI), collectively known as Gulf War illness (GWI) [1]. Prevalent complaints are central nervous system (CNS) dysfunction, musculoskeletal pain, fatigue, respiratory symptoms, gastrointestinal issues, immunological dysfunction, and skin problems. Specific patterns of medical/cognitive symptoms have been characterized to be associated with GWI and CMI [2,3,4], and these symptoms have been consistently reported in US, UK, and Australian GW veterans (GWVs) [5] and persist 30 years after their return from the Gulf [6,7].

Central nervous symptom disorders are prevalent in GWVs, with elevated rates of amyotrophic lateral sclerosis (ALS), stroke, migraine headaches, neuralgia/neuritis, and brain cancer [8,9,10]. In addition, recent evidence suggests that a substantial number of GWVs have reported mild and other traumatic brain injuries over their life course (m/TBI; [11,12,13]), which is a risk factor for pathological aging/dementia [14] and chronic traumatic encephalopathy (CTE; [15]). Gulf War veterans also exhibit neurobehavioral deficits, such as memory problems, frontal system dysfunction, slowed motor and processing speeds, sustained attention deficits, reduced visuospatial skills and psychomotor dysfunction [8,16,17].

Although the causes of these disorders remain elusive, several environmental exposures have been implicated as potential contributors to altered CNS function, including exposure to acetylcholinesterase (AChE) inhibitors, such as pyridostigmine bromide (PB; antinerve gas pills) and organophosphate (OP) pesticides/nerve agents (e.g., sarin/cyclosarin) [18]. Thus, GWI and associated disorders may be related to whether GWVs were exposed to OP nerve agents (e.g., destruction of the munitions depot in Khamisiyah, Iraq, during the war) as well as their premorbid vulnerability (e.g., genetic) to such exposures. The association of GW deployment to GWI and CNS disorders will require ongoing observation of GWVs to elucidate potential causes.

Mouse models, induced pluripotent stem cells (iPSCs) from GWVs with GWI, and other methods have been used to study potential pathophysiological mechanisms, but there is a critical need for research-quality human CNS tissue to advance genomic and proteomic research on GWI and related neurological disorders. The Gulf War Veterans’ Illnesses Biorepository (GWVIB), part of the VA Biorepository Brain Bank (VABBB), supports this critical need as a CNS tissue biorepository that conducts extensive antemortem longitudinal assessments on enrolled GWVs with subsequent postmortem CNS tissue recovery. Data and tissue maintained in the GWVIB are available to investigators studying GWI and related disorders.

## 2. Materials and Methods

### 2.1. Project Overview

**Figure 1:**
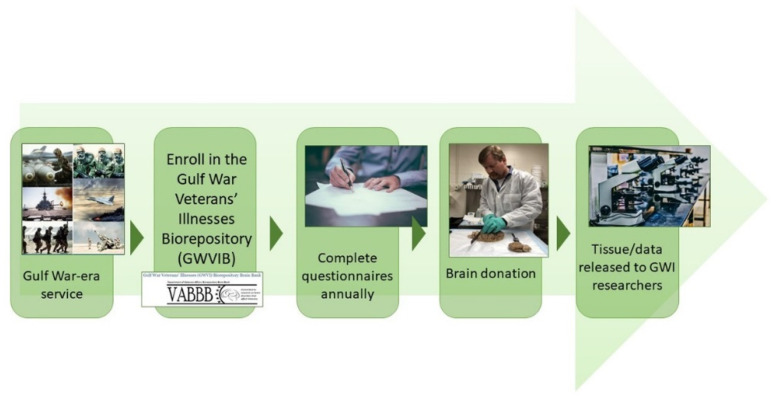
Gulf War Veteran’s Pathway to Participation in the Gulf War Veterans’ Illness Biorepository.

The GWVIB is a national prospective cohort study (see Figure 1 for an overview). The CNS tissue bank began in 2015 and enrolls veterans who served during the 1990–1991 Gulf War era, also known as Operation Desert Shield/Storm, regardless of whether a veteran was deployed to the Gulf during the conflict (i.e., all GW era veterans). The GWVIB conducts annual follow-up telephone and mail interviews to collect health, cognition, and other data until the veteran’s passing, when the veteran’s brain and spinal cord are recovered for expedited shipment to the biorepository. Study coordination, participant enrollment/follow-up and diagnostic neuropathology are conducted at the VA Boston Healthcare System (VABHS). Tissue is processed and stored at the Southern Arizona VA Healthcare System (SAVAHCS) in Tucson, AZ, USA. Tissue requests and disbursements for research are coordinated by SAVAHCS. Any researcher (VA, non-VA/academic, or commercial) examining GWI and related disorders may apply for tissue and data. Tissue request procedures may be found on the “For Investigators” tab at https://www.research.va.gov/programs/tissue_banking/gwvib/default.cfm. The GWVIB complies with VA human subjects and biorepository regulations. Human subject regulatory oversight for the Boston site is via the Institutional Review Board and Research and Development Committee at VABHS, with data/biological repository regulatory oversight for the Tucson site via the SAVAHCS Research and Development Committee. The GWVIB is funded and overseen by the VA Gulf War Research Program.

### 2.2. Participants

#### 2.2.1. Recruitment

Gulf War era veterans are recruited nationally by VABHS staff via the VA Informatics and Computing Infrastructure (VINCI) Veterans Health Administration (VHA) Corporate Data Warehouse (CDW), social media, press releases, other Gulf War illness studies, and our website (https://www.research.va.gov/programs/tissue_banking/gwvib/default.cfm). Recruitment through VINCI is via a search of the CDW for potential GWV participants to distribute recruitment materials, including introductory letters, brochures, and permission to contact (PTC) forms via mail and email. The GWVs interested in participating return the signed PTC forms by mail/secure fax/email or contact the GWVIB by telephone.

#### 2.2.2. Enrollment

VABHS staff contact interested GWVs and consent those who wish to enroll either in person (in the Boston area) or via mail/secure email or telephone depending on the enrollee’s situation. The enrollee’s legal next of kin (NOK) also cosigns a provisional consent agreeing to the future postmortem donation during the enrollment process because the NOK must approve the donation upon the veteran’s passing. A postmortem consenting procedure via telephone and secure fax/email is used in cases where the veteran expressed interest in enrolling but died before granting consent.

### 2.3. Study Procedures

#### 2.3.1. Antemortem Assessments

VABHS staff call the enrolled veteran and/or their next of kin (in cases where the veteran cannot speak) to conduct an initial baseline assessment. A brief cognitive assessment is given to GWVs who can speak (described below). Veterans enrolled in the New England area are asked to give a voluntary one-time blood sample (21 mL) prior to death at a time convenient to them. A baseline health questionnaire is also mailed to the veteran to complete and return. During all subsequent annual follow-up calls, staff update the medical history/cognition assessments. Relevant medical history data are also extracted from the VA electronic medical record (EMR) through the Compensation and Pension Record Interchange (CAPRI). The data are downloaded/transferred with appropriate permissions.

#### 2.3.2. Blood, Brain, and Spinal Cord Recovery; Processing; Tissue Quality Assessments; and Neuropathology

Antemortem blood samples are shipped to SAVAHCS for processing. Plasma is collected by centrifugation, and peripheral blood mononuclear cells (PBMCs) are isolated and frozen down in liquid nitrogen. RNA and DNA from PAXgene tubes are extracted and frozen down at −80 °C and made available for future release to investigators. Because polymorphisms (L55M and Q192R) in *paraoxonase 1 (PON1)* have been associated with the development of neurologic symptom complexes in GWVs, we assess these polymorphisms in all samples. Annotating our samples prior to storage expedites the future release of blood samples to investigators seeking to study the role of *PON1* in GWI. Due to the aging of the cohort and the established relation of *Apolipoprotein E* (*APOE)* genotype to dementia risk (which may confound *PON1* findings), we also determine *APOE* genotype (ε2, ε3, or ε4) [19].

Upon being notified of an enrollee’s passing, an on-call VABHS researcher contacts the veteran’s next of kin to reconfirm the donation authorization and coordinates tissue recovery. SAVAHCS initially receives the postmortem CNS tissue donation (i.e., brain/spinal cord/pituitary) for processing. Postmortem specimens are processed as frozen or formalin-fixed specimens following modified procedures outlined by Vonsattel et al. and others [20,21,22]. Upon specimen receipt, the pituitary and olfactory bulbs/nerves are identified and placed in 10% formalin solution for fixation. Abnormal pituitary regulation in association with the hypothalamic–pituitary–adrenal axis (HPA) has been associated with GWI as well as other chronic conditions [23,24,25]. The whole cerebral brain, cerebellum, and brainstem are hemisected at the midsagittal plane. Right and left portions are alternated by their ID number to designate which hemisphere will be used for preparation of formalin-fixed or liquid-nitrogen-frozen specimens (e.g., even # = left hemisphere). One cerebral hemisphere with attached hemi-brainstem and cerebellum is immersed in 10% formalin. The hemi-brainstem and cerebellum are separated from the other hemisphere. The cerebellum half is sectioned sagittally (0.5–1 cm thick) prior to freezing, and the bisected brainstem (midbrain, pons, and medulla oblongata) is transversely sectioned (3–5 mm thick) for freezing. The cerebral hemisphere is then cut in sequential 0.5–1 cm thick coronal slices for preparation of liquid-nitrogen-frozen specimens. After thorough examination of all half-brain coronal slices and digital imaging with measurements, the slices are frozen in sequential anterior to posterior order. Because of the diversity of brain anatomic regions considered to be affected in GWI, specific frozen coronal regions will later be selected and dissected on dry ice depending on an investigator’s request and defined experimental purposes. Moreover, depending on our own neuropathological findings, this process is revised to include more detailed dissection and archiving at the time of fresh specimen grossing. Based on GWI brain imaging reports, cerebral regions of research interest may include specific basal ganglia (caudate, putamen, subthalamic nucleus, etc.), hippocampus, amygdala, thalamus, and prefrontal and gray/white matter domains (for examples, see [26,27,28,29,30,31,32,33]). The spinal cord is sectioned transversely (3–5 mm thick) with alternate sections from each region (cervical, thoracic, and lumbar) either frozen or fixed in formalin. Prior to archiving at −80 °C, all frozen specimens are carefully 2D bar-coded and catalogued in the study database. Frozen occipital lobe cortex is primarily used for evaluations of RNA integrity and brain tissue pH. Tissue pH is determined as previously described [20,34,35]. All formalin-fixed specimens are sent overnight by certified shipment to VABHS for further tissue processing and neuropathological review. 

All cases are subsequently neuropathologically evaluated at VABHS for age-related and disease-specific pathologies. Normal cases from veterans with no known neurological or psychiatric disorders undergo a similar confirmation process. Tissue blocks are taken and paraffin embedded from 34 regions. Histochemistry and immunohistochemistry are performed on select blocks as previously described [36]. All cases are evaluated fully for neurodegenerative disease, and an extensive report with gross and microscopic examination results as well as semiquantitative interpretation of the immunohistochemical data are generated. We perform assessments of vascular disease, Braak and Braak staging for neurofibrillary tangles, Braak staging and NIA-AA classification [37] for Lewy bodies, CERAD staging and Thal phasing for Aβ, and evaluation for TDP-43 pathology [38,39] and age-related tau astrogliopathy [40]. Neurodegenerative evaluations are compatible with the National Alzheimer’s Coordinating Center requirements [41]. These data are entered into a Research Electronic Data Capture (REDCap) database for future retrieval and analysis. The strength of this approach involves the pathological confirmation and characterization of various neurodegenerative diseases as well as the collection of semiquantitative data available for clinicopathological studies. Brain tumors are pathologically evaluated using current WHO diagnostic criteria [42]. This includes IDH-1 mutation status using immunohistochemistry or sequencing when warranted. All cases are reviewed at a monthly consensus conference held with Boston and Tucson staff to fully characterize the neuropathological findings, integrate phenotype data, and approve the case for future data/tissue releases.

#### 2.3.3. Specimen Disbursement to Investigators

Any researcher (VA, non-VA/academic, or commercial) examining GWI and related disorders may apply for tissue and data. Tissue request procedures may be found on the “For Investigators” tab at https://www.research.va.gov/programs/tissue_banking/gwvib/default.cfm. Tissue requests are reviewed by a Tissue Access Committee. Approval criteria are scientific merit, experimental design and biostatistics, supporting data, investigator qualifications, and tissue availability. Investigators and their institutional officials as well as officials at VABHS/SAVAHCS and VA Office of Research and Development must sign a Material Transfer/Data Use Agreement before specimens are released. 

Specimens are provided as histological slides (fixed or frozen) or frozen fragments. For immunohistochemistry projects, one FFPE slide (Luxol/H&E stained) is provided per case per tissue region. Investigators receive 3–5 charged glass slides per case per CNS region of 4–6 µm thick FFPE tissue sections. Tissue slides can be used for immunohistochemistry, in situ hybridization, or microdissection. Fragments from topographically specific sites may be requested for molecular or PCR testing in sections no greater than 100 mg. Associated case data are provided with the specimens, including gender, age, health (including GWI-specific assessments) and cognitive assessments, postmortem intervals, tissue quality (RIN and tissue pH based on occipital lobe), neuropathology diagnosis, and other data if approved by the Tissue Access Committee. Tissue is shipped in compliance with all relevant national and international shipping regulations.

### 2.4. Measures

#### 2.4.1. GWVIB Baseline Survey

The GWVIB baseline survey is composed of questions from other measures as detailed below and items developed by the GWVIB in collaboration with other GWI studies.

Structured Neurotoxicant Assessment Checklist—Short Form (SNAC; [43]). It has been found in several studies of GWI that those with higher exposure to pesticides and other neurotoxicants have increased symptoms, including higher rates of CMI [8,44]; accordingly, we document past exposures to environmental hazards. The SNAC asks the veteran to report approximate number of days they were exposed to potential toxicants, including tent heaters, pesticides, DEET, delousers, smoke from oil well fires, pest strips, and nerve agent antidote (pyridostigmine bromide). In addition, postdeployment occupational and recreational use of potential neurotoxicants is listed, and the veteran is asked to document the amount and type of exposures to chemicals such as lead solders, lead paints, degreasers, and other solvents that may impact brain function. 

Symptom questions used to identify Gulf War illness by Kansas case definition and chronic multisymptom illness by Centers for Disease Control (CDC) case definition. We utilize symptom questionnaires developed by Steele [3] and Fukuda [2] as criteria for GWI and CMI, respectively. The Kansas and CDC criteria inquire about specific medical symptoms and their severity are used throughout clinical and research settings as an index of the respective Gulf War related disorders. The Kansas GWI definition includes exclusionary criteria for conditions that may confound self-reporting of GWI-specific symptoms. GWVs with these conditions were excluded from the case definition.

The Veterans RAND 12-Item Health Survey (VR-12; [45]). The VR-12 is a brief (adapted from the longer VR-36), self-administered 12-item health survey primarily used to measure physical and mental health status and related quality of life. The 12 items are summarized into two scores, namely a physical component score (PCS) and a mental health component score (MCS), which provide a contrast between physical and psychological health status. We followed the PCS and MCS summary score calculation in which scores are standardized using a t-score transformation that is normed to a US population with a score of 50 and a standard deviation of 10 [46]. Higher values reflect better physical- and mental health related quality of life.

Alcohol, Smoking, and Substance Involvement Screening Test (ASSIST; [47]). The ASSIST was developed by the World Health Organization to assess current and lifetime alcohol, tobacco, and psychoactive substance use and related problems. We used a reduced item set to assess current and/or past use of these substances. Items related to intervention were not administered as participants do not receive interventions as a result of this assessment.

#### 2.4.2. Cognitive Interview

Telephone Interview for Cognitive Status—Modified (TICSm; [48,49,50]; with additional tests). The TICSm is a brief telephone cognitive assessment similar to the Mini-Mental State Exam (MMSE; [51]) that assesses orientation, concentration, memory (immediate and short-delay word list recall), responsive naming, comprehension, calculation, reasoning, and judgment. The maximum score is 50, and a score of 27 or below is the usual cutoff for possible cognitive impairment [52,53]. The test has been well validated [48,49,54]. We added a forced-choice recognition trial after the short-delay recall of the 10-item word list to further assess aspects of forgetfulness (e.g., encoding vs. retrieval deficits in memory). Additional tests are administered to better assess attention, working memory, and frontal systems functions posited to be affected in GWI [55,56]. An adapted Digit Span Forward and Backward from the Wechsler Adult Intelligence Scale [57] is given by administering one trial rather than the traditional two trials at each span. The forward span measure is an index of attention, whereas the backward span measure is an index of working memory [58]. Verbal fluency is used as an executive function measure where enrollees name as many different animals as possible in 60 s.

## 3. Results

### 3.1. Participants/Study Population

We received the contact information for 365,135 potential GWVs from the VINCI CDW search, and as of September 2021, a GWVIB recruitment packet has been mailed to about 4500 veterans across all 50 states and the District of Columbia. To date, 131 GWVs were consented into the GWVIB; 113 are living enrollees currently being followed, 14 have passed away and donated, 2 passed away and declined donation, and 2 withdrew from follow-up and donation. Of the 127 enrolled GWVs from 39 states, approximately two-thirds have come from our VINCI mail recruitment, with the remainder from the other recruitment methods. The cohort has been followed for up to six years thus far. 

Table 1 lists the baseline characteristics of 96 of the 127 currently enrolled GWVs. The 31 without baseline data had either not yet completed enrollment assessments (27) or opted to participate via medical record review/data collection with postmortem donation only (4). Fifty-four enrollees were deployed in support of the 1990–1991 Gulf War. The other GWVs who reported that they were either not deployed to the Gulf or were not deployed in support of the Gulf War are important Gulf War era control participants. Many of the 96 GWVs had myriad military-related exposures (e.g., medical, environmental, and combat), including 12 who were (and another 12 unsure if they were) notified of sarin/cyclosarin exposure at Khamisiyah, Iraq. Fourteen met the criteria for GWI (39 other GWVs were excluded from the case definition per Kansas criteria) and 60 for CMI. Further, substantial proportions of GWVs reported head trauma and comorbid health disorders (e.g., sleep disorder, posttraumatic stress disorder, and memory loss). Scores on the VR-12 show that the average MCS score (45.1) was similar to the population average of 50; however, the PCS score (36.6) was nearly 1.5 standard deviations below 50, suggesting that this GWV cohort was endorsing substantially worse physical quality of life compared with their mental quality of life. Performance on the TICSm on average (35.5) was above the cutoff of 27.

### 3.2. Tissue Donor Inventory, Quality, and Diagnostic Neuropathology

Table 2 lists the characteristics of GWV tissue donors and tissue quality metrics. As of September 2021, the GWVIB recovered 14 cases in seven states. Six donors met the criteria for CMI, and two met the criteria for GWI. The average age of death was 65 (range 54 to 75). Postmortem interval averaged 34 h with a range of 22 to 65 h. To characterize specimens for future applications, parameters of brain pH and RNA integrity (as depicted by RIN values) were assessed. Postmortem brain pH is reflective of the antemortem agonal state and is considered an important factor influencing RNA integrity [35,59,60]. All cases had RIN values ≥4.8 with a mean of 5.8 (SD = 1.0). 

Table 2 also shows the neuropathological diagnoses made in six cases that have undergone neuropathological analyses thus far. Three cases had an ALS diagnosis related to the initial history of the GWVIB co-enrolling GWVs from the pre-existing VABBB ALS brain bank. Notably, given the substantial rate of head trauma reported in our cohort, we also found that two of the three ALS cases had comorbid chronic traumatic encephalopathy (CTE). Further, other notable neuropathological conditions in our cohort were Lewy body disease, glioblastoma, and Alzheimer’s disease. The other eight recovered GWVs are currently undergoing neuropathology review. The neuropathology seen in the cohort thus far is consistent with that reported in the GWI research literature (e.g., [9,61,62]) and suggests that our recruitment efforts are producing a cohort to support research on relevant disorders affecting GWVs. 

## 4. Discussion

To our knowledge, the GWVIB is the only ongoing prospective observational cohort study and CNS tissue biorepository focused on disorders affecting GWVs. Though still early in the collection of the cohort for study, the current cohort is broadly representative of the Gulf War related exposures and disorders reported by GWVs. Over half were deployed to the Gulf, and substantial numbers of those enrolled have experienced myriad medical, environmental, and combat exposures, including potential sarin/cyclosarin exposure at Khamisiyah, Iraq, which may be related to CNS changes in GWVs [13,33,63,64]. Additionally, two-thirds of those assessed met the criteria for CMI and/or GWI, and the cohort reported substantially lower physical versus mental quality of life on the VR-12. Further, 34 veterans were not deployed in support of the Gulf War and represent an important Gulf War era nondeployed control group for comparison.

Six of the 14 recovered cases met the criteria for CMI or GWI. Neuropathology seen in six of the recovered cases analyzed thus far has been consistent with CNS disorders reported in the GWI/CMI literature (e.g., [9,61,62]. Because veterans are at increased risk of traumatic brain injury and repetitive head impacts, including blast exposure, which are risk factors for CTE, it is perhaps unsurprising that CTE was one of most frequent pathologies found thus far in our deceased GWVs. This pattern has been shown in our separate ALS brain bank as well [61]. The other eight cases are currently undergoing neuropathological review.

Given the depth and breadth of our data and the initial findings in our tissue collection from GWVs, the GWVIB is poised to support current and future research on GWI/CMI and related disorders affecting GWVs. For example, studies have begun to link OP exposures in the Gulf to neurocognitive deficits and reduced brain volumes seen in GWV [13,33,64,65,66]. Accordingly, our assessment of GW-related exposures (e.g., Khamisiyah weapons site exposures) will allow researchers to examine possible sequalae of exposures such as these in postmortem CNS tissue. 

Generalizations between our study and others are limited in that we enrolled a veteran cohort that is largely a convenience sample. Furthermore, a substantial portion of cohort was largely enrolled from the CDW VINCI database, which contains only veterans who are enrolled in the VA healthcare system, and may not represent the larger GWV population. Moreover, selection bias may have been introduced because our cohort is a motivated sample interested in participating in an observational CNS biorepository without a treatment component. Finally, our neuropathology results are very limited due to the small number of GWVs who have donated and undergone neuropathological analyses. These limitations should lessen as the GWVIB continues and increases its enrollment and tissue resources.

Research on the etiology and treatment of GWI/CMI and related CNS disorders is dependent on ongoing collaborative longitudinal observational and interventional studies [1,67]. Despite the limitations described above, the GWVIB is uniquely positioned to address these critical needs as a CNS tissue and data biorepository to support national and international GWI research. Furthermore, forming collaborations between the GWVIB and other GWI study cohorts (e.g., [68]) to allow co-enrollment of GWVs will add substantial scientific value to all GWI research via the rich longitudinal data and tissue resources that the GWVIB can provide to other GWI studies at a relatively low incremental cost. Information about the GWVIB and tissue/data request procedures may be found at https://www.research.va.gov/programs/tissue_banking/gwvib/default.cfm

## 5. Conclusions

The potential causes of GWI/CMI and related disorders continue to be examined 30 years after GWVs returned from the Gulf. Environmental and other exposures during Gulf War deployment may be related to CNS changes seen in GWVs, and these CNS changes may play a role in the development of GWI/CMI and related disorders. The GWVIB, an ongoing national prospective cohort study and CNS tissue bank maintains a repository of health data and postmortem CNS tissue from GWVs with GWI/CMI and relevant controls. The GWVIB data and tissue repository is a unique and important resource for investigators studying the role of CNS dysfunction in Gulf War-related disorders. Continuing enrollment of GWVs into the GWVIB will enhance the value of the repository. Tissue and data are available from the GWVIB for request by any researchers conducting research on disorders affecting Gulf War veterans. 

## Figures and Tables

**Table 1:** Characteristics of the GWVIB cohort.

Baseline Characteristics of the GWVIB Cohort (*n* = 96 *)	
Characteristic	
Current age, mean (SD), y	64.3 (7.8)
Age range, y	49–84
Gender (#)	
Men	79
Women	17
Ethnicity/race (#)	
Not Spanish, Hispanic, or Latino	80
White	78
African American	4
American Indian/Alaska Native	3
Other	11
Education (#)	
High school/GED	5
Some college	20
Associates degree	12
Bachelor’s degree	24
Master’s degree	19
Professional or Doctorate degree	7
Military branch ^1^ (#)	
Army	53
Navy	17
Air Force	14
Marines	11
Coast Guard	1
Military service era (#)	
September 2001 or later	31
August 1990 to August 2001 (includes Gulf War)	96
May 1975 to July 1990	76
August 1964 to April 1975 (Vietnam era)	24
Serve outside US? (#)	78
Where were you stationed? (#)	
Africa	9
Asia/South Pacific	37
Caribbean	9
Eastern Europe	7
Middle East	49
Northern/Central Europe	45
Southern Europe/Mediterranean Basin	16
South Central America	17
USA/Canada	64
Deployed in support of the 1990–1991 Gulf War (#)	54
Notified of Khamisiyah exposure, Yes/unsure (#)	12/12
Military exposures (#)	
Combat exposure	40
Blast exposure	13
Pyridostigmine bromide pills (sure/unsure)	32/13
Chemical/biological warfare agents (sure/unsure)	22/36
Anthrax vaccine (sure/unsure)	43/16
Agent orange (sure/unsure)	5/18
Health history (#)	
Concussion with loss of consciousness in lifetime	33
Traumatic brain injury diagnosis—ever	8
Memory loss diagnosis—ever	18
Sleep disorder—ever	49
Posttraumatic stress disorder diagnosis—ever	32
Cancer diagnosis—ever	19
Chronic multisymptom illness (CMI)—CDC criteria (#)	60
Gulf War Illness (GWI)—Kansas criteria (#) ‡	14
Veterans RAND 12-Item Health Survey, mean (SD)	
Physical component score (PCS)	36.6 (12.6)
Mental component score (MCS)	45.1 (13.9)
Telephone Interview of Cognitive Status—Modified, mean (SD)	35.5 (4.8)

Note. * Values based on 96 of 127 enrollees with data available in September 2021. ^1^ Some enrollees served in multiple military branches. ‡ # Of those cases who met inclusion criteria for consideration of GWI; 39 were excluded per Kansas criteria.

**Table 2:** Characteristics of the GWVIB tissue donors.

Characteristics of the Gulf War Veteran Tissue Donors (*n* = 14 *)	
Characteristic	
Age at death, mean (SD), y	65.2 (6.6)
Men, #	13
Racial/ethnic group, #	
White, non-Hispanic	13
Hispanic	1
Chronic multisymptom illness (CMI)—CDC criteria (#)	6
Gulf War illness (GWI)—Kansas criteria (#) ‡	2
Neuropathological diagnoses, # * ^1^	
ALS	2
ALS/FTLD	1
Glioblastoma	1
Lewy body disease	2
CTE	2
AD	1
Other ^2^	6
PMI, mean (SD), range, h	34.4 (14.1), 22–65
RIN, mean (SD), range	5.8 (1.0), 4.8–7.8
pH, mean (SD), range	6.3 (0.4), 5.9–6.9

Note: Causes of death: respiratory complications due to ALS, non-ALS related respiratory failure, complications due to cancer, aspiration pneumonia, suicide. * Neuropathological diagnoses available on 6/14 donors in September 2021. Abbreviations: ALS = amyotrophic lateral sclerosis, FTLD = frontotemporal lobar degeneration, AD = Alzheimer’s disease, CTE = chronic traumatic encephalopathy, PMI = postmortem interval, RIN = RNA integrity number, pH = potential of hydrogen. ^1^ Each case has more than one diagnosis. ^2^ Other diagnoses include primary age-related tauopathy, limbic-predominant age-related TDP-43 encephalopathy, cerebral amyloid angiopathy, stroke, athero/arteriosclerosis. ‡ # Of those cases who meet inclusion criteria for consideration of GWI.

## Data Availability

The data presented in this study are available on request via a Data Use Agreement from the corresponding author. The data are not publicly available due to Department of Veterans Affairs data safety and privacy regulations.
